# *Rac*-GR24-Induced Defense Responses Against Gray Mold in Postharvest Cherry Fruit Revealed by Physiological and Omics Analyses

**DOI:** 10.3390/foods15213801

**Published:** 2026-10-23

**Authors:** Huihui Wang, Yifan Zhu, Ze Li, Xiaoan Li, Huihui Xu, Shanshan Hao, Aqleem Abbas, Yanyin Guo, Nana Ji

**Affiliations:** College of Agricultural Engineering and Food Science, Shandong University of Technology, Zibo 255049, China; wanghh0510@163.com (H.W.);

**Keywords:** sweet cherry, strigolactone, postharvest, gray mold, resistance, omics analyses

## Abstract

Strigolactones (SLs) are carotenoid-derived phytohormones that regulate plant growth, development, and stress adaptation. Although SL-related regulation of plant immunity has been increasingly recognized, its role in postharvest disease resistance remains largely unexplored. In this study, postharvest cherries were treated with different concentrations (0, 0.01, 0.1, 0.5, 1, 1.5, and 2 μM) of *rac*-GR24, a synthetic SL analogue, or with Tis-108, a chemical inhibitor of SL biosynthesis that targets the cytochrome P450 MAX1 (CYP711A), prior to inoculation with *Botrytis cinerea*. Among the tested concentrations, 0.1 μM *rac*-GR24 most effectively suppressed gray mold development, as reflected by reduced disease incidence, lesion diameter, and fungal biomass compared with the control, together with enhanced activities of defense-related enzymes, including CHI and GLU, whereas Tis-108 treatment aggravated disease development. Integrated transcriptomic and metabolomic analyses revealed significant changes in pathways related to phenylalanine, tyrosine, and tryptophan biosynthesis, as well as phenylpropanoid and flavonoid biosynthesis. Notably, *rac*-GR24 treatment markedly increased the expression and activities of key enzymes involved in the phenylpropanoid pathway, including PAL, C4H, and 4CL. Consistent with these molecular responses, *rac*-GR24 treatment significantly increased the accumulation of total phenolics, flavonoids, and representative compounds such as catechin, epicatechin, rutin, and chlorogenic acid. Overall, these findings suggest that *rac*-GR24 treatment is associated with defense responses and changes in phenylpropanoid metabolism in postharvest sweet cherry. This study provides new insights into the molecular basis of defense responses induced by *rac*-GR24 and highlights its potential as an effective strategy to reduce postharvest losses.

## 1. Introduction

Sweet cherry (*Prunus avium* L.) is highly valued by consumers for its attractive appearance, desirable flavor, nutritional value, and health-promoting properties [1]. However, it is a highly perishable fruit owing to its thin epidermis, high moisture content, and active metabolism after harvest, all of which contribute to rapid quality deterioration during storage and distribution [2]. Among postharvest pathogens, *Botrytis cinerea* is an important causal agent of gray mold in sweet cherry, causing fruit rot and substantial postharvest losses [3,4]. The extensive use of chemical fungicides for postharvest disease control may result in pesticide residues on fruit, raising concerns regarding food safety and potential risks to human health [5].

Phytohormones are increasingly recognized as promising, environmentally friendly regulators of plant defense responses and postharvest quality [6]. Plants activate complex adaptive responses under stress, which are coordinated by interconnected phytohormonal networks that modulate defense-related pathways and systemic acquired resistance (SAR) [7]. Several phytohormones, including salicylic acid (SA), jasmonates (JAs), abscisic acid (ABA), and ethylene (ET), participate in the regulation of disease resistance in fruits and have been implicated in responses to postharvest pathogens [8,9,10,11,12].

SLs are carotenoid-derived phytohormones that play important roles in plant growth, development, and stress adaptation. In canonical SL biosynthesis, all-trans-β-carotene is sequentially converted by D27, CCD7, and CCD8 to produce carlactone, which is subsequently oxidized by MAX1 and related cytochrome P450 enzymes to generate SLs, including 5-deoxystrigol, 4-deoxyorobanchol, and orobanchol [13]. SLs have been implicated in diverse physiological processes, including shoot branching, seed germination, leaf senescence, adventitious root formation, stress responses, and disease resistance [14,15,16,17,18,19]. Recent studies have also begun to reveal potential beneficial effects of SL-related treatments in postharvest fruits and vegetables. For example, 1.0 μM SL application positively maintained fruit quality in postharvest strawberry fruit by enhancing antioxidant metabolism and phenylpropanoid biosynthesis [20]. Similarly, 200 μM SL-treated sweet orange fruit exhibited delayed senescence and quality deterioration [21]. Additionally, SL improves tolerance to chilling stress in tomato, litchi, and zucchini fruits during cold storage [22,23,24]. Owing to the extremely low endogenous levels of natural SLs, the synthetic SL analogue *rac*-GR24 has been widely used as a tool to investigate SL-related physiological responses [25].

Tis-108 is a chemical inhibitor commonly used to interfere with SL biosynthesis, and its application has been used to investigate the potential involvement of SL-related processes in plant responses [26]. *Rac*-GR24 and Tis-108 are widely used to study SL functions in tomato [27], olive [28], and strawberry [20] with evidence showing their effects on hormone homeostasis and transcriptional regulation [29]. However, research on the disease resistance induced by SLs has mainly focused on model plants such as *Arabidopsis* and tomato, with relatively few studies examining their effects on postharvest disease resistance in fruits, particularly in perennial woody plants like cherry.

Hence, this study aimed to determine an effective concentration of *rac*-GR24 for reducing *B. cinerea* infection in postharvest sweet cherry and to characterize the molecular and metabolic responses associated with its application. Integrated transcriptomic and metabolomic analyses were conducted to characterize molecular and metabolic responses associated with *rac*-GR24 treatment, with particular emphasis on phenylpropanoid and flavonoid biosynthesis. The results provide new insights into the defense responses associated with *rac*-GR24 treatment and highlight its potential as a sustainable approach for reducing postharvest gray mold in sweet cherry.

## 2. Materials and Methods

### 2.1. Fruit and Treatments

Sweet cherry fruit (*Prunus avium* L. cv. Xianfeng) was harvested at commercial maturity from a commercial orchard in Huantai District, Zibo City, Shandong Province, China. Fruits with uniform color and size and free from visible defects or mechanical damage were selected and immediately transported to the laboratory within 2 h and acclimated at 20 ± 1 °C for 2 h to dissipate field heat.

*B. cinerea* was obtained from the China General Microbiological Culture Collection Center (CGMCC, Beijing, China) and cultured on Potato Dextrose Agar (PDA) medium (Qingdao Hope Bio-Technology Co., Ltd., Qingdao, China). The culture was harvested and suspended in sterile water containing 0.5% Tween-80 (Shanghai Macklin Biochemical Co., Ltd., Shanghai, China) to prepare a spore suspension at a concentration of 1.0 × 10^5^ spores mL^−1^. *Rac*-GR24 (CAS No. 76974-79-3; purity > 98%; Solarbio Science & Technology Co., Ltd., Beijing, China) was evaluated at concentrations ranging from 0 to 2 μM in a preliminary concentration–response experiment. Tis-108 (CAS No. 1315459-30-3; purity > 99.6%; Shanghai Yuanye Bio-Technology Co. Ltd, Shanghai, China) was used at 5 μM as a chemical inhibitor commonly used to interfere with SL biosynthesis.

The *rac*-GR24 concentration applied in this research was selected based on a preliminary dose–response assay (0, 0.01, 0.1, 0.5, 1, 1.5, and 2 μM). Cherry fruits were surface-disinfected with 2% NaClO (Xilong Scientific Co., Ltd., Shantou, China) for 2 min, rinsed with tap water for 30 s, and then air-dried [30]. The fruits were randomly assigned to three treatment groups: water control, 0.1 μM *rac*-GR24, and 5 μM Tis-108 [31], and immersed in the corresponding solutions for 7 min at 20 ± 1 °C. After drying, one uniform wound (2 mm diameter × 2 mm depth) was made on the equatorial region of each fruit, followed by a 6 h equilibration period and inoculation with 10 μL of *B. cinerea* spore suspension. The inoculated fruits were incubated at 20 ± 1 °C, and lesion diameter and disease incidence were assessed at 12, 24, 36, 48, and 60 h. The experiment included six sampling times: 0 h (before treatment) and 12, 24, 36, 48, and 60 h after treatment. Each treatment included 960 fruits, divided into three independent biological replicates of 320 fruits each. At each sampling time point, 60 fruits per treatment were used and randomly divided into three groups of 20 fruits each, with each group serving as one biological replicate. The pooled tissues were immediately frozen in liquid nitrogen and stored at −80 °C until further analyses.

### 2.2. Lesion Diameter and Disease Incidence

Lesion diameter was measured using a digital Vernier caliper at each sampling time point. Sweet cherries were recorded as diseased when the lesion diameter was >3 mm at the inoculation site. Disease incidence was calculated as the percentage of infected wounds relative to the total number of inoculated wounds.

### 2.3. Chitinase (CHI) and β-1,3-Glucanase (GLU) Activities

CHI and GLU activity were measured as described by Abeles [32], with minor modifications. Cherry samples were homogenized in pre-chilled 0.1 M sodium acetate buffer (pH 5.0; sodium acetate and glacial acetic acid from Macklin Biochemical Co., Ltd., Shanghai, China) using a SCIENTZ-48LD high-throughput frozen grinder (Ningbo Scientz Biotechnology Co., Ltd., Ningbo, China) and centrifuged at 12,000× *g* for 20 min at 4 °C (TGL-20M; Hunan Xiangyi Laboratory Instrument Development Co., Ltd., Changsha, China). The resulting supernatant was then collected for the determination of CHI and GLU enzyme activities. One unit (U) of CHI activity was defined as the amount of enzyme required to increase the absorbance at 524 nm by 0.01 per minute. GLU activity was determined using the reducing sugar method with laminarin (Solarbio Science & Technology Co., Ltd., Beijing, China) as the substrate. One U of GLU activity was defined as the amount of enzyme that produced 1 mg of glucose per hour. Both activities were expressed as U g^−1^ fresh weight (FW). Absorbance was measured using a UV-1900i spectrophotometer (Shimadzu Corporation, Kyoto, Japan).

### 2.4. Fungal Biomass Assessment

To quantify the fungal biomass of *B. cinerea* in cherry pulp following inoculation, a DNA-based qPCR method was used to estimate the relative abundance of *B. cinerea*. Total DNA was extracted from infected cherry pulp using a genomic DNA extraction kit (9768S, Takara, Kusatsu, Japan) according to the manufacturer’s protocol, and no reverse transcription step was involved. The primers used for qPCR were designed based on the *B. cinerea Bccap1* gene sequence, which encodes a conserved F-actin-capping protein subunit alpha. This gene was selected as the target for DNA-based qPCR to estimate the relative abundance of fungal material in infected cherry tissues. The primer sequences are listed in Table S1 and were synthesized by Sangon Biotech (Shanghai) Co., Ltd. (Shanghai, China). qPCR was performed using a LightCycler 96 instrument (Roche Diagnostics, Basel, Switzerland) with 2× SYBR Green PCR Mastermix (Beijing Solarbio Science & Technology Co., Ltd., Beijing, China). Relative fungal biomass was calculated using the comparative Ct method according to the formula 2 ^ ^(CT(cherry EF1-α) − CT(Bccap1))^. This normalization approach is consistent with previously published methods [33].

### 2.5. Transcriptome and Metabolome Profiling

**Figure 1:**
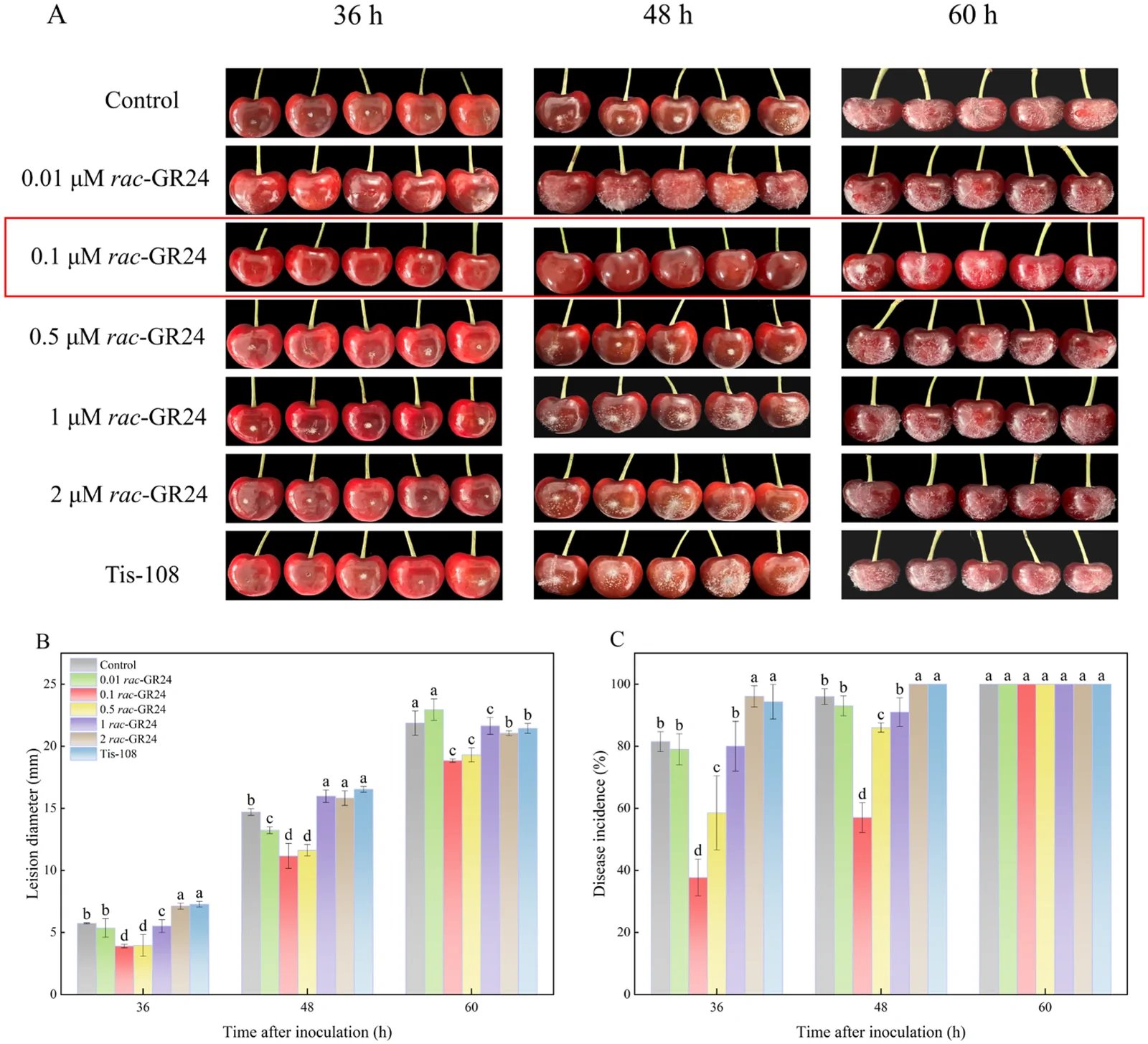
Effects of *rac*-GR24 and Tis-108 treatments on gray mold development in sweet cherry fruit. (**A**) Disease development, (**B**) lesion diameter, and (**C**) disease incidence; the figures show data only for infected samples. Fruits were wounded (2 mm in diameter and 2 mm in depth) and inoculated with 10 μL of *B. cinerea* spore suspension (1.0 × 10^5^ spores mL^−1^). The red box in (**A**) highlights the 0.1 μM rac-GR24 treatment, which showed the best effect among all tested concentrations. Each biological replicate consisted of 20 fruits, and three independent biological replicates were analyzed for each treatment. Data are presented as mean ± SD (*n* = 3). Different letters at the same time point indicate significant differences (*p* < 0.05).

Based on disease progression observations, significant differences among treatments became apparent between 24 and 48 h post inoculation (hpi) (Figure 1). Considering that early defense signaling activation and metabolic reprogramming generally occur before visible symptom development, samples collected at 0 h and 24 h were selected for transcriptomic and metabolomic analyses. For transcriptome sequencing and untargeted metabolomics, four groups were analyzed: Control 0 h, Control 24 h, Tis-108 24 h, and *rac*-GR24 24 h. For each group, tissues from 20 fruits were pooled to constitute one biological replicate, and three independent biological replicates were analyzed separately for each group. For each biological replicate, 10 g of the pooled tissue sample was used for omics analysis. The same pooled samples were used for both transcriptomic and metabolomic analyses. Transcriptome sequencing and untargeted metabolomics were performed by Shanghai Majorbio Bio-Pharm Technology Co., Ltd. (Shanghai, China). The transcriptome assembly was performed using StringTie, and the expression was quantified as FPKM (Fragments Per Kilobase of transcript per Million mapped reads) [34]. Differential expression analysis was subsequently conducted using the DESeq2 R package, where genes meeting the criteria of differential fold change > 2 with an adjusted *p*-value < 0.05 were identified as differentially expressed genes (DEGs) [35]. For metabolites, differentially accumulated metabolites (DAMs) were identified based on stringent criteria, including differential fold change > 2, variable importance in projection (VIP) scores > 1, and *p* < 0.05. Functional enrichment analysis of DEGs and DAMs was performed using Gene Ontology (GO) and Kyoto Encyclopedia of Genes and Genomes (KEGG) pathway enrichment analyses, and terms with an adjusted *p* < 0.05 were considered statistically significant.

### 2.6. Phenylalanine Ammonia Lyase (PAL), Cinnamate 4-Hydroxylase (C4H), and 4-CoumarateCoA Ligase (4CL) Activities

PAL activity was determined according to Assis [36], with one U defined as an absorbance increase of 0.01 at 290 nm per h. C4H activity was determined according to the method described by Lamb and Rubery [37]. Briefly, 1 g of frozen sample was homogenized in extraction buffer containing 50 mM Tris-HCl (pH 8.9; Solarbio Science & Technology Co., Ltd., Beijing, China), 5 mM ascorbic acid (Shanghai Macklin Biochemical Co., Ltd., Shanghai, China), 15 mM βmercaptoethanol (Shanghai Macklin Biochemical Co., Ltd., Shanghai, China), 4 mM MgCl_2_ (Shanghai Macklin Biochemical Co., Ltd., Shanghai, China), and 10% glycerol (Tianjin Zhiyuan Chemical Reagent Co., Ltd., Tianjin, China) using a chilled mortar and pestle. One U of C4H activity was defined as the amount of enzyme required to increase the absorbance at 340 nm by 0.01 per hour under standard assay conditions.

4CL activity was determined according to the established spectrophotometric method of Knobloch and Hahlbrock [38], with one U of enzyme activity defined as the quantity catalyzing an absorbance increase of 0.01 per hour at 333 nm under standard assay conditions. All enzyme activities were expressed as U g^−1^ FW.

### 2.7. Proanthocyanidins and Lignin Contents

Proanthocyanidin content was determined using the p-dimethylaminocinnamaldehyde (DMAC) colorimetric method [39], based on the reaction mechanism in which DMAC undergoes acid-catalyzed dehydration condensation with flavan-3-ol units of proanthocyanidins, yielding a blue-colored chromophore exhibiting maximum absorbance at 640 nm. The absorbance intensity exhibits a linear correlation with analyte concentration over a defined range, allowing for precise spectrophotometric determination.

Lignin content was measured using a commercial assay kit (CAS, Solarbio Science & Technology Co., Ltd., Beijing, China) following the manufacturer’s protocol. The phenolic hydroxyl groups in lignin were acetylated, generating a characteristic absorption peak at 280 nm. The absorbance at 280 nm was positively correlated with lignin content. The results are expressed as mg g^−1^ FW.

### 2.8. Phenolic and Flavonoid Contents

Frozen cherry samples were homogenized in 80% (*v*/*v*) pre-chilled acetone (Sinopharm Chemical Reagent Co., Ltd., Shanghai, China) and centrifuged at 4 °C. The supernatant was collected for quantification of total phenolic and flavonoid contents. Total phenolic content was quantified using the Folin–Ciocalteu (Solarbio Science & Technology Co., Ltd., Beijing, China) method provided by Slinkard [40]. The supernatant was reacted with Folin–Ciocalteu and Na_2_CO_3_ (Shanghai Macklin Biochemical Co., Ltd., Shanghai, China), and then incubated at 30 °C for 30 min in the dark, and the absorbance was measured at 765 nm. Quantification was performed using a gallic acid (Solarbio Science & Technology Co., Ltd., Beijing, China) standard curve, with results expressed as milligrams of gallic acid equivalents per kilogram of fresh weight (mg GAE/kg FW).

Total flavonoid content was quantified using the aluminum chloride colorimetric assay described by Toor [41], with rutin (Solarbio Science & Technology Co., Ltd., Beijing, China) as the calibration standard. Absorbance was measured at 430 nm after 30 min incubation with the reaction mixture (containing 1 mL 30% AlCl_3_ [Shanghai Macklin Biochemical Co., Ltd., Shanghai, China] and 0.5 mL 70% ethanol [Sinopharm Chemical Reagent Co., Ltd., Shanghai, China]) at room temperature, and the result was expressed as milligrams rutin equivalent per kilogram of fresh weight (mg rutin/kg FW).

Individual phenolic compounds were quantified according to the method reported by Ceccarelli, with minor modifications [42]. Analysis was carried out using an L Agilent 1260 Infinity HPLC system (Agilent Technologies, Santa Clara, CA, USA). Chromatographic separation was achieved on a reversed-phase C18 column (250 mm × 4.6 mm) (InfinityLab Poroshell 120; Agilent Technologies, Santa Clara, CA, USA) maintained at 30 ± 0.5 °C. The column temperature was maintained at 30 °C. The mobile phase flowed at 0.8 mL min^−1^, with the detection wavelength set at 280 nm, and the injection volume was 20 μL. Methanol (5%; Tedia High Purity Solvents, Fairfield, OH, USA) and acetonitrile (Tedia High Purity Solvents, Fairfield, OH, USA) served as mobile phases A and B, respectively. Quantification analysis was performed using established calibration curves with concentrations expressed as mg g^−1^ FW.

### 2.9. RT-qPCR Analysis

To validate transcriptomic data, eleven representative genes involved in pathogen defense and phenylpropanoid metabolism were selected: Chitinase, β-1,3-glucanase, pathogenesis-related protein 4, phenylalanine ammonia lyase, Cinnamate 4-hydroxylase, 4-coumarate-CoA ligase, chalcone synthase, dihydroflavonol 4-reductase, cinnamyl-alcohol dehydrogenase, and peroxidase. Specific primers were designed based on sequences obtained from the NCBI database (Table S1). Total RNA was extracted following Chang [43], and cDNA synthesis was performed using the ToloScript All-in-One RT Easy Mix Kit (Tolobio, Shanghai, China). The relative gene expression was calculated using the 2^−ΔΔCt^ method with EF1-α as the internal reference gene. The cherry *EF1-α* gene was used to normalize for variations in the amount of total DNA extracted from each sample, since the same amount of tissue was used for DNA extraction across all samples.

### 2.10. Statistical Analysis

The experiment consisted of three treatments (water, 0.1 μM *rac*-GR24, and 5 μM Tis-108). Samples were collected at 0 h before treatment and at 12, 24, 36, 48, and 60 h after treatment and inoculation. For each biological replicate, tissues from the lesion margins of 20 fruits were pooled to generate one composite sample, and three independent biological replicates (*n* = 3) were collected for each treatment × time combination. The 20 fruits within each composite sample were considered technical replicates and were averaged prior to analysis; they were not treated as independent experimental units. Because the primary objective was to compare treatments at each sampling time, one-way ANOVA followed by Duncan’s multiple range test (*p* < 0.05) was performed separately at each time point using SPSS 27.0 (IBM, Armonk, NY, USA). Pearson’s correlation coefficients were calculated to assess the relationships between physiological and transcriptomic parameters, and correlation diagrams were visualized using Origin 2021 (OriginLab Corporation, Northampton, MA, USA). A probability value of *p* < 0.05 was considered statistically significant. Each set of experiments was performed in triplicate, and data are presented as means ± standard deviation (SD).

## 3. Results

### 3.1. Optimal Rac-GR24 Concentration for Disease Control

Preliminary experiments were conducted to determine the optimal *rac-GR24* concentration for disease control. Among the various *rac-GR24* treatments tested (0.01, 0.1, 0.5, 1.0, 1.5, and 2.0 μM), low to moderate concentrations (0.01–0.5 μM) effectively reduced postharvest disease in cherry compared to the control group, and the most pronounced disease resistance was observed after 0.1 μM *rac-GR24* treated (highlighted by the red box in Figure 1A; Figure 1A–C). In contrast, higher concentrations (1.0, 1.5, and 2.0 μM) unexpectedly accelerated disease lesions (Figure 1B,C). Based on these results, 0.1 μM *rac-GR24* was identified as the optimal concentration and selected for all subsequent experiments.

### 3.2. Disease Progression in Cherry Fruit

**Figure 2:**
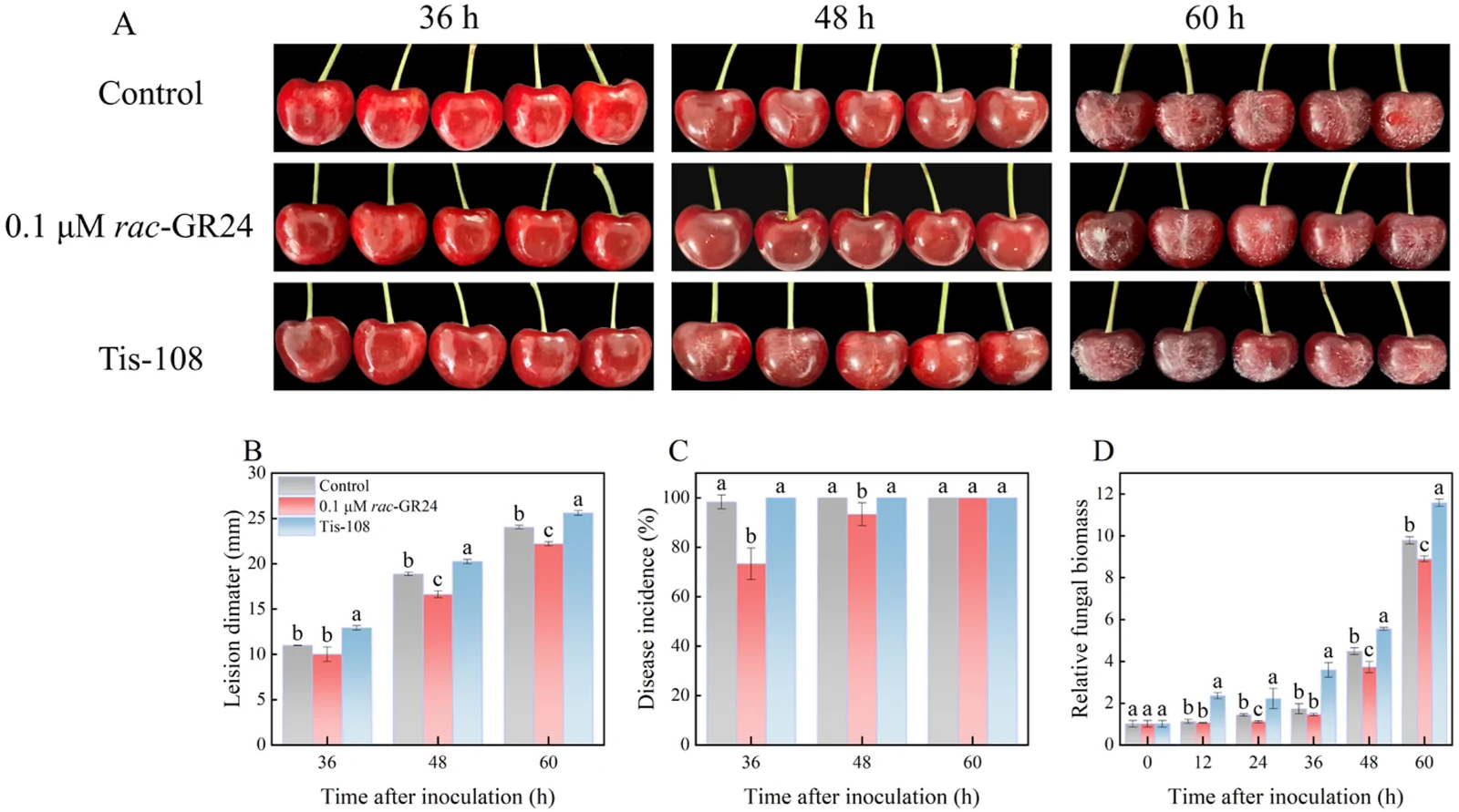
Effects of *rac*-GR24 and Tis-108 treatments on gray mold development and fungal biomass in sweet cherry fruit. (**A**) Disease development, (**B**) lesion diameter, and (**C**) disease incidence; the figures show data only for infected samples. (**D**) Relative fungal biomass. Fruits were wounded (2 mm in diameter and 2 mm in depth) and inoculated with 10 μL of *B. cinerea* spore suspension (1.0 × 10^5^ spores mL^−1^). Each biological replicate consisted of 20 fruits, and three independent biological replicates were analyzed for each treatment. Data are presented as mean ± SD (*n* = 3). Different letters at the same time point indicate significant differences (*p* < 0.05).

Following *B. cinerea* inoculation, disease development in all treatments was consistent with preliminary observations (Figure 2A). After inoculation, lesion diameter, disease incidence, and relative fungal biomass increased in all three treatment groups (Figure 2B–D). However, the *rac*-GR24 treatment significantly suppressed these increases and effectively delayed pathogen proliferation, whereas Tis-108 accelerated the disease progression.

### 3.3. Transcriptional and Enzymatic Changes in Chitinase and β-1,3-Glucanase

**Figure 3:**
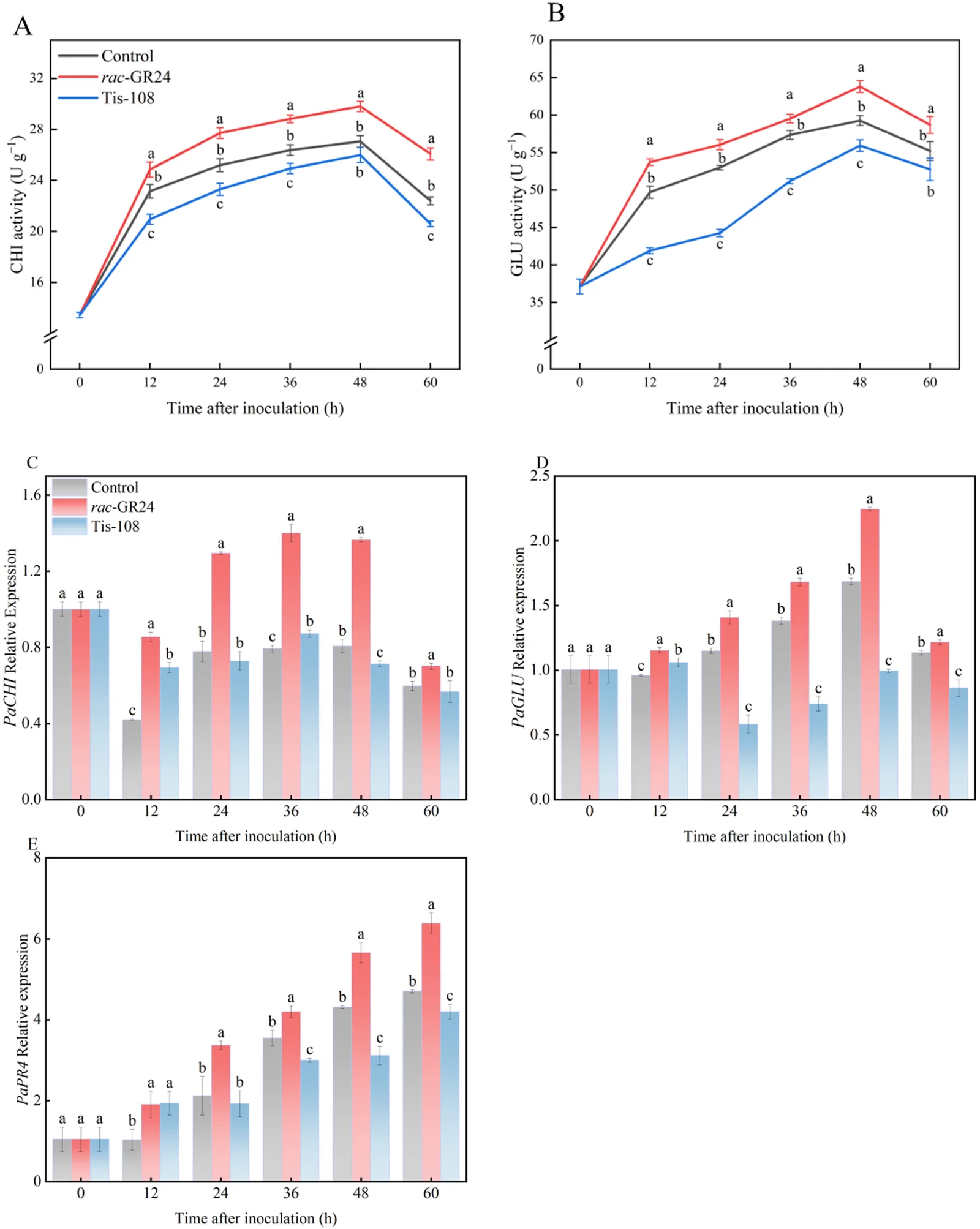
Changes in enzymatic activities and expression levels of CHI (**A**,**C**) and GLU (**B**,**D**) in cherry fruit and *PaPR4* relative expression (**E**). Fruits were wounded (2 mm in diameter and 2 mm in depth) and inoculated with 10 μL of *B. cinerea* spore suspension (1.0 × 10^5^ spores mL^−1^). Each biological replicate consisted of 20 fruits, and three independent biological replicates were analyzed for each treatment. Data are presented as mean ± SD (*n* = 3). Different letters at the same time point indicate significant differences (*p* < 0.05).

As shown in Figure 3, *rac*-GR24 treatment simultaneously enhanced the activities of CHI and GLU, together with the expression of *PaCHI* and *PaGLU* during the first 48 h of inoculation, with the most pronounced differences observed at 36 and 48 h, at which point they increased by 1.40-fold and 2.24-fold, respectively, followed by a gradual decline. The transcript level of *PaPR4* increased progressively throughout inoculation, a trend similar to that of CHI and GLU, and was also induced by *rac*-GR24 but suppressed by Tis-108.

### 3.4. Integrated Transcriptomic and Metabolomic Analysis

The reliability of the transcriptome analysis was demonstrated by the high-quality reads, accurate sequence alignment, high mapping rate, and transcript assembly, as shown in Table S2.

**Figure 4:**
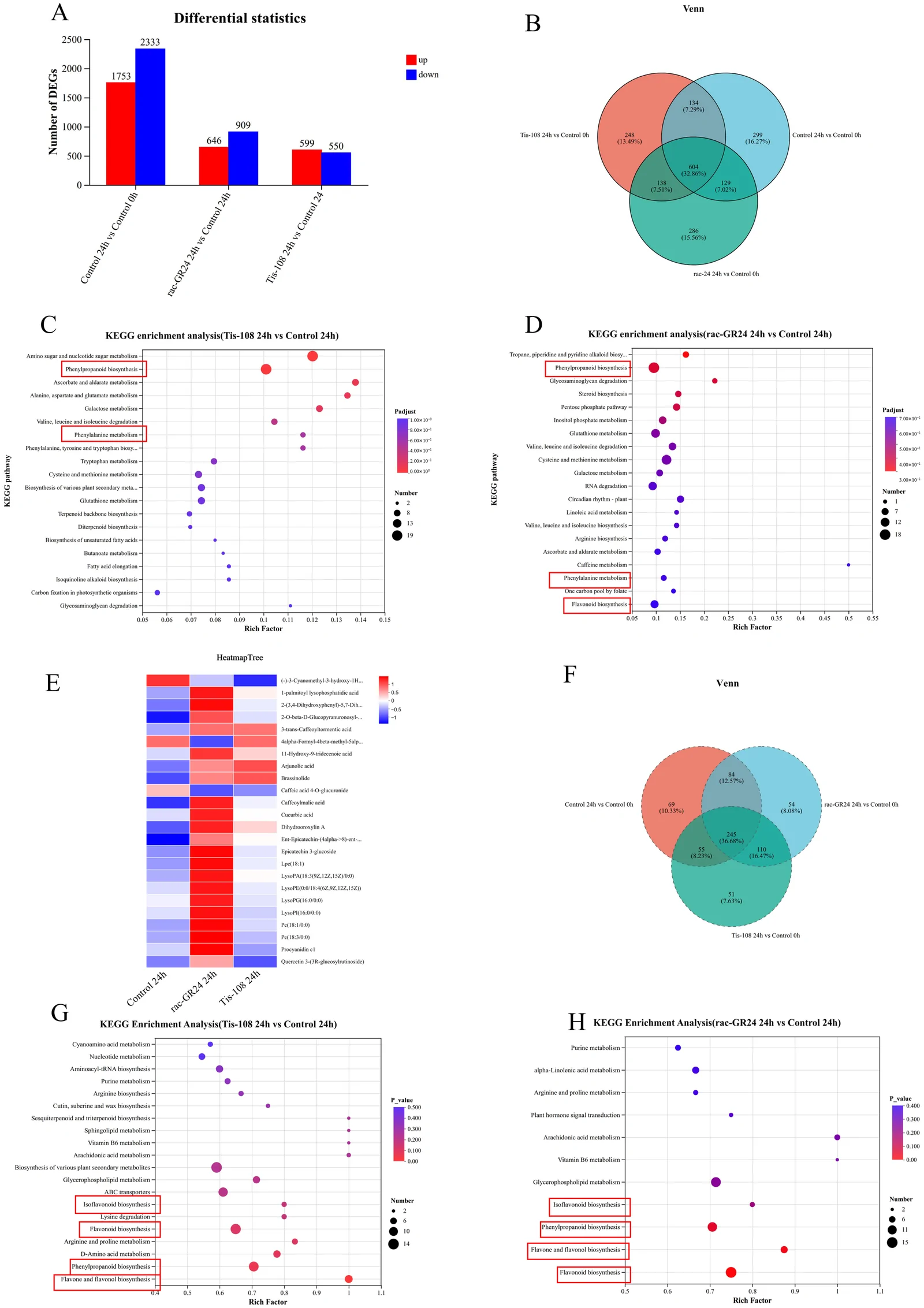
Analysis of DEGs and DAMs in cherry fruit of groups: Control, *rac*-GR24 and Tis-108 after inoculation. (**A**) Number of upregulated and downregulated DEGs in each pairwise comparison. (**B**,**F**) Venn diagrams showing shared and unique DEGs and DAMs. (**C**,**G**) KEGG pathway enrichment analysis of DEGs and DAMs: Tis-108 24 h vs. Control 24 h. (**D**,**H**) KEGG pathway enrichment analysis of DEGs and DAMs: *rac*-GR24 24 h vs. Control 24 h. (**E**) Heatmap of metabolite cluster analysis. Fruits were wounded (2 mm in diameter and 2 mm in depth) and inoculated with 10 μL of *B. cinerea* spore suspension (1.0 × 10^5^ spores mL^−1^). Red boxes highlight the pathways focused on in this analysis. For molecular analyses, tissues from the 20 fruits within each replicate were pooled to generate one composite sample.

Pairwise comparisons identified DEGs, including 1753 upregulated and 2333 downregulated genes in Control 24 h vs. Control 0 h, 646 upregulated and 909 downregulated genes in *rac*-GR24 24 h vs. Control 24 h, and 599 upregulated and 550 downregulated genes in Tis-108 24 h vs. Control 24 h (Figure 4A). Venn analysis showed 604 genes shared among all treatments, suggesting a role in fundamental physiological processes during fruit development (Figure 4B). In addition, 742 genes were commonly regulated by both *rac*-GR24 and Tis-108. KEGG pathway analysis revealed that the DEGs in the comparisons of Tis-108 24 h vs. Control 24 h and *rac*-GR24 24 h vs. Control 24 h were both enriched in Phenylpropanoid biosynthesis and Phenylalanine metabolism (Figure 4C,D). A total of 54 DEGs from these pathways were subsequently selected for further analysis (Table S3).

Metabolomic analysis further identified 245 shared DAMs across all treatments, which may contribute to physiological processes during fruit growth and development (Figure 4F). Comparative analysis revealed that metabolites in *rac*-GR24 24 h vs. Control 24 h and Tis-108 24 h vs. Control 24 h were significantly enriched in phenylpropanoid biosynthesis, flavonoid biosynthesis, isoflavonoid biosynthesis, and flavone and flavonol biosynthesis (Figure 4G,H). Notably, the coordinated changes observed at the transcriptional and metabolic levels were associated with the activation of phenylpropanoid and flavonoid metabolism following *rac*-GR24 treatment. These findings suggest that changes in these pathways may contribute to the enhanced postharvest disease resistance observed in *rac*-GR24-treated cherry fruit.

### 3.5. Transcriptome and Metabolome Analysis of Phenylpropane Metabolism

**Figure 5:**
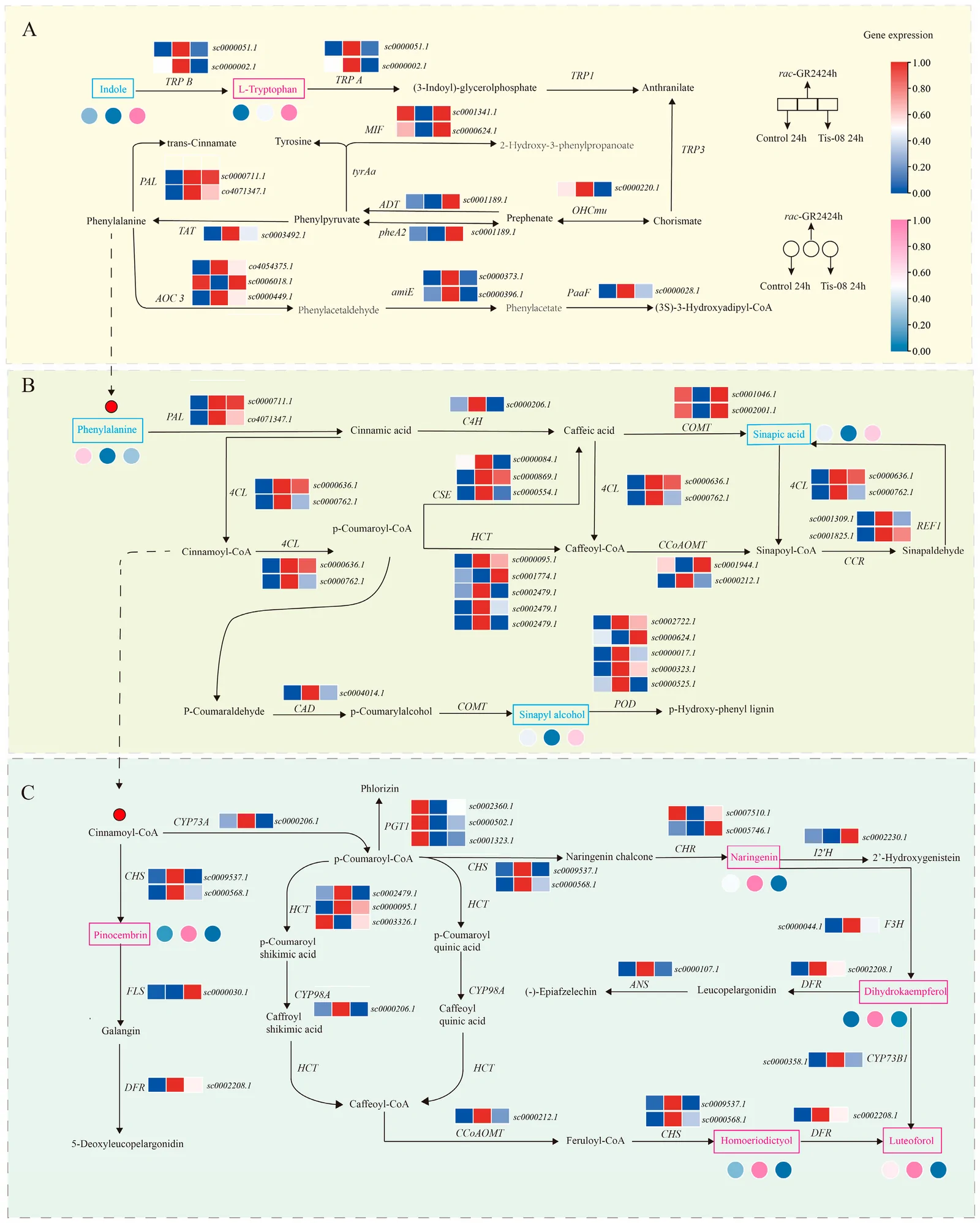
Integrated transcriptomic and metabolomic analysis of DEGs and DAMs associated with (**A**) phenylalanine, tyrosine, and tryptophan biosynthesis; (**B**) phenylpropanoid biosynthesis and (**C**) flavonoid biosynthesis in cherry fruit 24 hpi. The rectangular color scale gradient from red to blue represents increasing FPKM values, and the circle color gradient from red to blue represents increasing metabolite abundance. Fruits were wounded (2 mm in diameter and 2 mm in depth) and inoculated with 10 μL of *B. cinerea* spore suspension (1.0 × 10^5^ spores mL^−1^). Arrows indicate the flow of metabolites. For molecular analyses, tissues from the 20 fruits within each replicate were pooled to generate one composite sample.

As shown in Figure 5A, a total of 18 DEGs were identified in phenylalanine biosynthesis. (Figure 5A). Compared with the control, *rac*-GR24 treatment significantly upregulated the expression of two PALs, *tryptophan synthase alpha chain* (*TRP A*) and *tryptophan synthase beta chain* (*TRP B*) genes, and one *aminotransferase TAT2 isoform X1* (*TAT*), *chorismate mutase* (*OHCmu*), *amidase* (*amiE*) and *enoyl CoA hydratase mitochondrial isoform X1* (*PaaF*) gene in cherry fruit. After 24 h of inoculation, two *copper amine oxidase 1-like genes* (*AOC3*, *co4054375.2*, *sc0000449.1*) were markedly upregulated by *rac*-GR24 treatment, whereas one *AOC3* gene (*sc0006018.1*), two *macrophage migration inhibitory factor homolog* (*MIF*) genes, one *arogenate dehydratase* (*ADT*) gene, and one *prephenate dehydratase* (*pheA2*) gene were significantly downregulated. In contrast, these genes were significantly upregulated under Tis-108 treatment. Among all the DEGs, most exhibited opposite expression trends in response to *rac*-GR24 and Tis-108 treatments, with the exception of *PAL*.

Metabolomic profiling revealed two DAMs, indole and L-tryptophan, associated with phenylalanine metabolism. As shown in Figure 5A, *rac*-GR24 treatment significantly reduced indole accumulation and consequently increased L-tryptophan levels compared with the control. In contrast, Tis-108 treatment led to a marked increase in both indole and L-tryptophan accumulation.

### 3.6. Transcriptome and Metabolome Analysis of Phenylpropanoid Biosynthesis

A total of 25 DEGs associated with phenylpropanoid biosynthesis were identified (Figure 5B), including five *peroxidase* (*POD*) and *shikimate O-hydroxycinnamoyltransferase* (*HCT*) genes; three *caffeoylshikimate esterase* (*CSE*) genes; two *PAL*, *4CL*, *caffeic acid 3O-methyltransferase* (*COMT*), *caffeoyl-CoA O-methyltransferase* (*CCoAOMT*), and *aldehyde dehydrogenase* (*REF1*) genes; and one *C4H* and one *cinnamyl alcohol dehydrogenase* (*CAD*) gene. Except for two *COMT* genes and *HCT* (*sc0001774.1*), *POD* (*sc0000624.1*), and *CCoAOMT* (*sc0001944.1*), all other genes showed higher expression levels under *rac*-GR24 treatment than in the control (Figure 5B). Phenylalanine, sinapic acid, and sinapyl alcohol were identified as DAMs associated with phenylpropanoid biosynthesis pathway, were significantly decreased by *rac*-GR24 treatment compared with the control. All identified DEGs and DAMs involved in the phenylpropanoid biosynthesis pathway exhibited opposite expression and accumulation trends between *rac*-GR24 and Tis-108 treatments, with the exception of *4CL* and *CSE* expression levels, together with the content of phenylalanine.

### 3.7. Transcriptome and Metabolome Analysis of Flavonoid Biosynthesis

Several key genes in the flavonoid biosynthesis pathway were identified (Figure 5C), including *trans-cinnamate 4-monooxygenase* (*CYP73A*, *sc0000206.1*), *chalcone synthase 5* (*CHS*, *sc0009537.1*, *sc0000568.1*), *HCT* (*sc0002479.1*, *sc0000095.1*), *leucocyanidin oxygenase* (*ANS*, *sc0000107.1*), *trans-cinnamate 4-monooxygenase* (*CYP98A*, *sc0000206.1*), *naringenin 3-dioxygenase* (*F3H*, *sc0000044.1*), *dihydroflavonol 4-reductase* (*DFR*, *sc0002208.1*), *flavonoid 3′-monooxygenase* (*CYP73B1*, *sc0000358.1*), *isoflavone 2′-hydroxylase-like isoform X1* (*I2′H*, *sc0002230.1*) and *CCoAOMT* (*sc0000212.1*). Compared to the control, *rac*-GR24 treatment significantly upregulated the expression of these genes (Figure 5C). In contrast, *rac*-GR24 treatment downregulated the expression of *anthocyanidin 5,3-O-glucosyltransferase-like* (*PGT1*, *sc0002360.1*, *sc0000502.1*, *sc0001323.1*), *flavonol synthase* (*FLS*, *sc0000030.1*), and *CHR* (*sc0007510.1*, *sc0005746.1*) genes at 24 hpi compared to the control treatment. Furthermore, *rac*-GR24 treatment promoted the accumulation of key flavonoid intermediates, including dihydrokaempferol, luteoforol, homoeriodictyol, naringenin and pinocembrin at 24 hpi. In contrast, Tis-108 exerted opposite regulatory effects on the expression of these genes and metabolites mentioned above compared with *rac*-GR24 except for the expression of *PGT1*.

### 3.8. Relative Expression Levels of MAX1, SMXL6, and SMXL7

**Figure 6:**
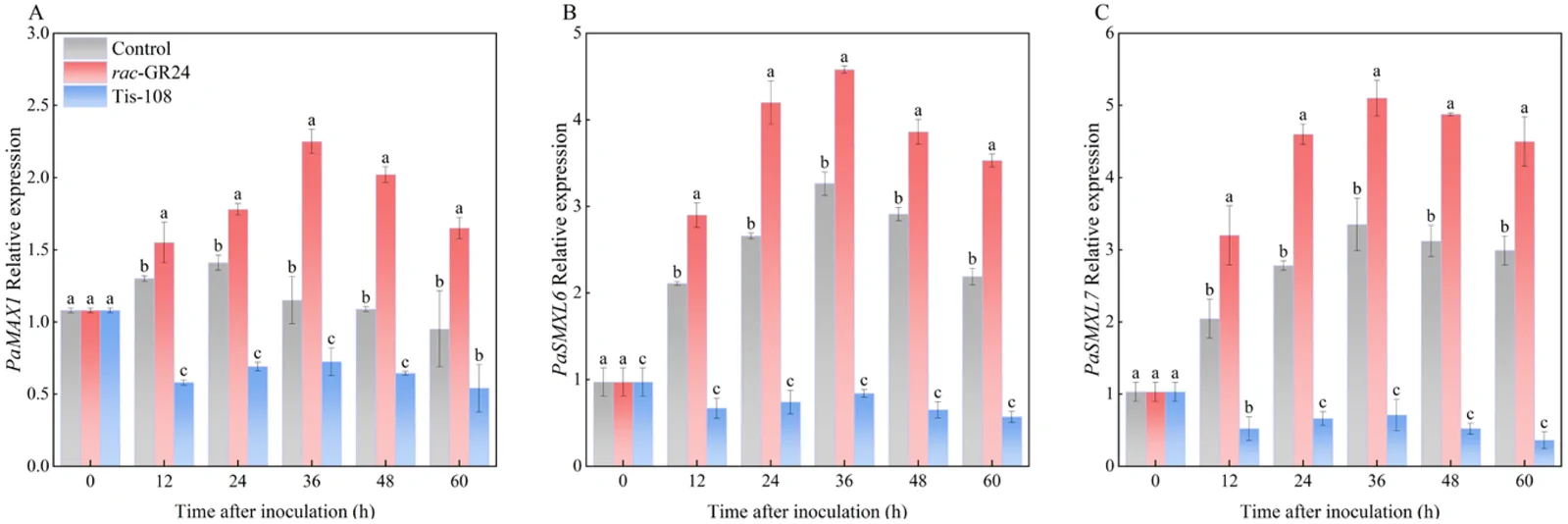
Relative expression levels of (**A**) *PaMAX1*, (**B**) *PaSMXL6*, and (**C**) *PaSMXL7* under different treatments over time. Fruits were wounded (2 mm in diameter and 2 mm in depth) and inoculated with 10 μL of *B. cinerea* spore suspension (1.0 × 10^5^ spores mL^−1^). Each biological replicate consisted of 20 fruits, and three independent biological replicates were analyzed for each treatment. Data are presented as mean ± SD (*n* = 3). Different letters at the same time point indicate significant differences (*p* < 0.05).

As shown in Figure 6, *rac*-GR24 treatment significantly upregulated the expression of *PaMAX1*, which is inhibited by Tis-108, as well as the SL signaling genes *PaSMXL6* and *PaSMXL7*. In contrast, Tis-108 treatment resulted in nearly undetectable levels of *PaMAX1* and suppressed the expression of *PaSMXL6* and *PaSMXL7*.

### 3.9. Transcriptional and Enzymatic Changes Related to the Phenylpropanoid Pathway

**Figure 7:**
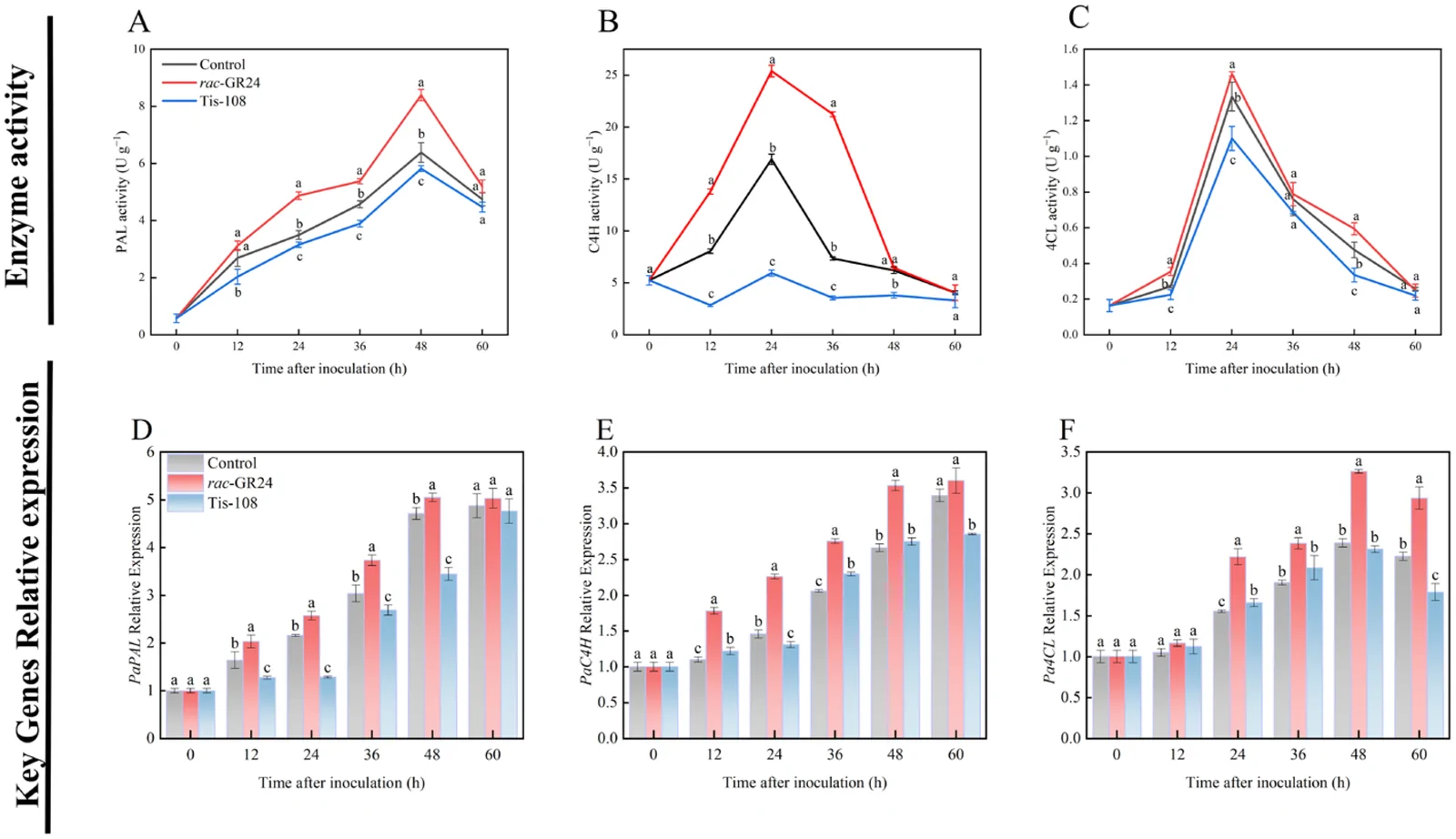
Activities and relative expression changes in (**A**,**D**) PAL activity, (**B**,**E**) C4H activity, and (**C**,**F**) 4CL activity in cherry fruit exposed to control, *rac*-GR24, or Tis-108 after inoculation. Fruits were wounded (2 mm in diameter and 2 mm in depth) and inoculated with 10 μL of *B. cinerea* spore suspension (1.0 × 10^5^ spores mL^−1^). Each biological replicate consisted of 20 fruits, and three independent biological replicates were analyzed for each treatment. Data are presented as mean ± SD (*n* = 3). Different letters at the same time point indicate significant differences (*p* < 0.05).

The activities of PAL, C4H, and 4CL showed a similar trend across treatments, characterized by early induction followed by gradual decline after inoculation (Figure 7). This response was markedly enhanced by *rac*-GR24. PAL activity peaked at 48 hpi, with *rac*-GR24-treated fruit showing a 1.59-fold increase compared with the control (*p* < 0.01) (Figure 7A). C4H activity reached its maximum earlier, at 24 hpi, and was 1.50-fold higher in the *rac*-GR24 group than in the control (*p* < 0.01) (Figure 7B). In contrast, 4CL activity varied less among treatments, although *rac*-GR24-treated fruit consistently maintained higher levels, peaking at 24 h and remaining significantly above both the control and Tis-108 groups throughout inoculation (Figure 7C).

The transcriptional profile of *PaPAL* exhibited a strong correlation with its enzyme activity pattern, showing an initial peak at 48 hpi, followed by a gradual decline (Figure 7D). The transcriptional profiles of *PaC4H* and *Pa4CL* showed strong correlations with their respective enzyme activity patterns, exhibiting an initial peak at 24 hpi, followed by progressive attenuation (Figure 7E,F).

### 3.10. Transcriptional and Enzymatic Changes Related to Lignin and Proanthocyanidin Biosynthesis and Accumulation

**Figure 8:**
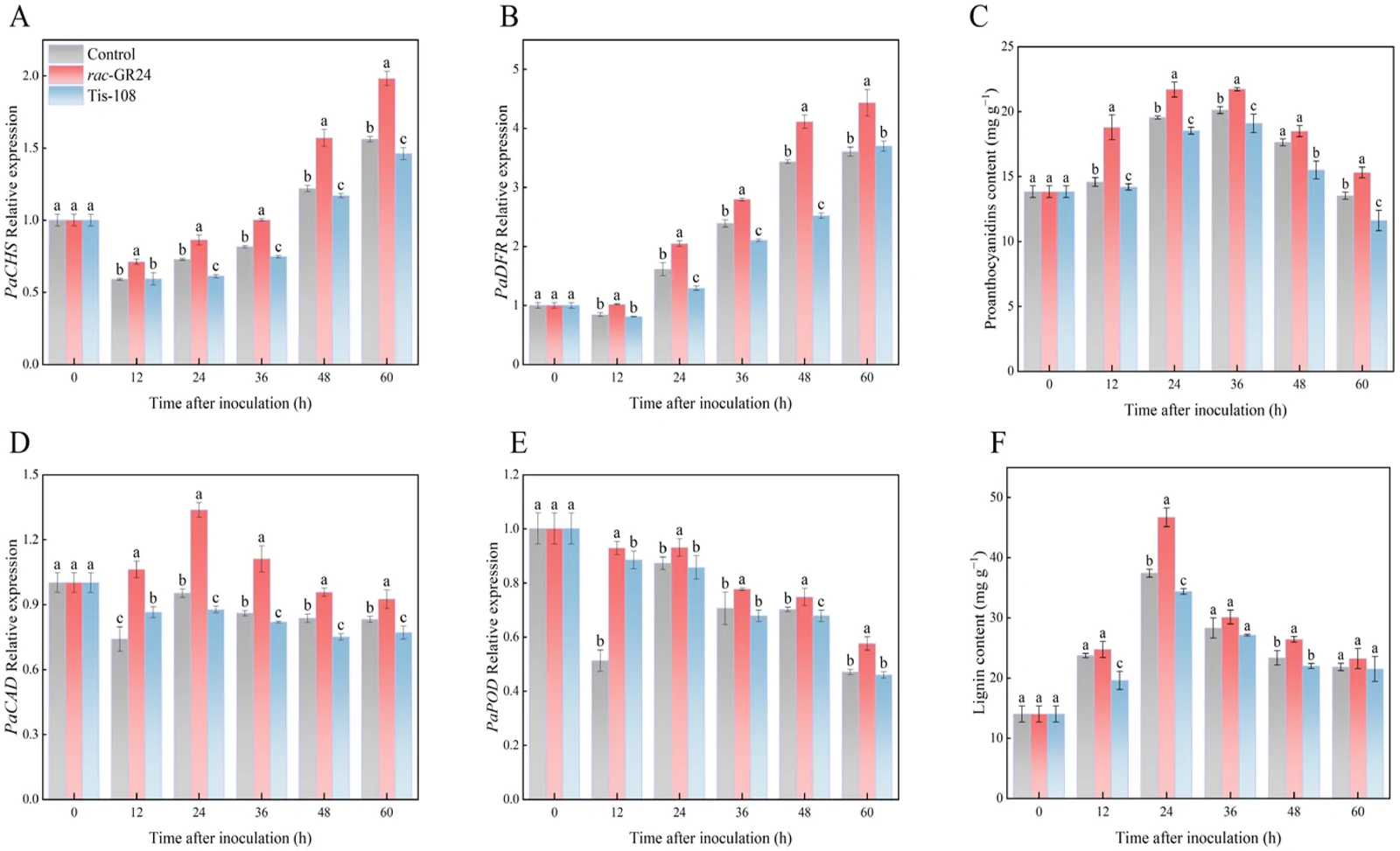
The relative expression changes in disease-resistant genes (**A**,**B**,**D**,**E**), *PaCHS*, *PaDFR*, *PaCAD* and *PaPOD*. Changes in the content of (**C**) proanthocyanins and (**F**) lignin. Fruits were wounded (2 mm in diameter and 2 mm in depth) and inoculated with 10 μL of *B. cinerea* spore suspension (1.0 × 10^5^ spores mL^−1^). Each biological replicate consisted of 20 fruits, and three independent biological replicates were analyzed for each treatment. Data are presented as mean ± SD (*n* = 3). Different letters at the same time point indicate significant differences (*p* < 0.05).

The accuracy of the transcriptomic data was validated by analyzing the transcript levels of four key genes (*PaCHS*, *PaDFR*, *PaCAD*, and *PaPOD*) and the contents of two key metabolites (proanthocyanidin and lignin). The expression of *PaCHS* showed a progressive increase, peaking at 60 hpi (Figure 8A). Correspondingly, the transcriptional profile of *PaDFR* was closely associated with proanthocyanidin accumulation, with expression levels under *rac*-GR24 treatment being significantly higher than those in the other two groups throughout the experimental period (Figure 8B). At the metabolic level, proanthocyanidin content in control fruit increased initially but declined thereafter, reaching its peak at 24 hpi (Figure 8C). In contrast, *rac*-GR24 treatment significantly promoted proanthocyanidin accumulation (*p* < 0.01), while Tis-108 consistently suppressed it below control levels throughout inoculation.

The relative expression level of *PaCAD* and *PaPOD* displayed an initial peak at 24 hpi, followed by a gradual decline (Figure 8D,E). Based on integrated transcriptomic and metabolomic analyses, the lignin biosynthesis pathway was significantly regulated (Figure 8B). *rac*-GR24 treatment enhanced the expression of key lignin biosynthesis genes (*PaCAD* and *PaPOD*; (Figure 8D,E), resulting in increased lignin accumulation (Figure 8F), whereas Tis-108 treatment had the opposite effect, suppressing both gene expression and lignin deposition. As shown in Figure 8, the expression patterns of these genes were highly consistent between RT-qPCR and transcriptomic data, confirming the reliability and validity of the transcriptomic analysis.

### 3.11. Contents of Main Individual Phenolic Compounds

**Figure 9:**
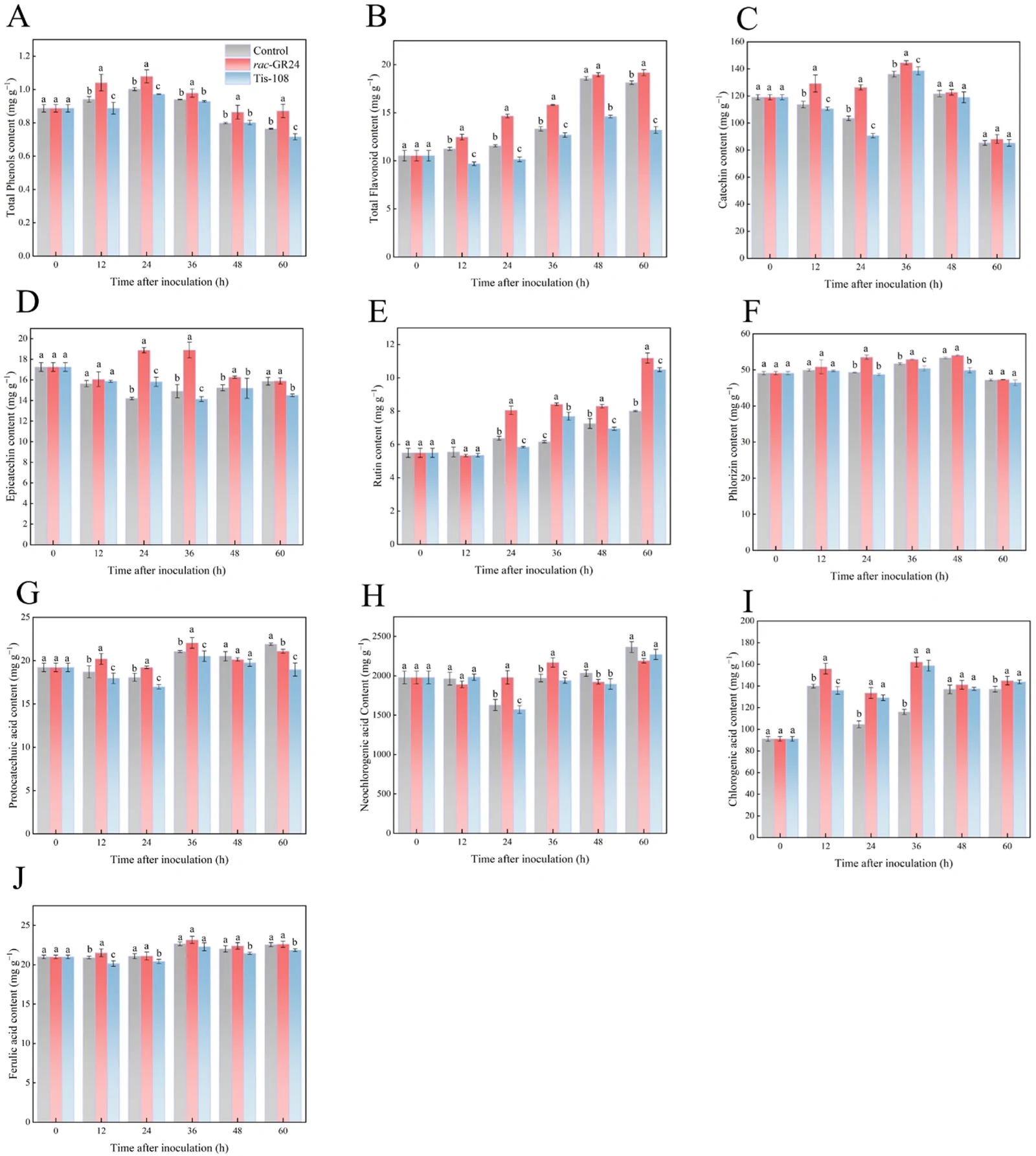
Changes in the contents of (**A**) total phenolics, (**B**) total flavonoids, (**C**) catechin, (**D**) epicatechin, (**E**) rutin, (**F**) phlorizin, (**G**) protocatechuic acid, (**H**) neochlorogenic acid, (**I**) chlorogenic acid and (**J**) ferulic acid in cherry fruit after treatment with control, *rac*-GR24 or Tis-108 after inoculation. Fruits were wounded (2 mm in diameter and 2 mm in depth) and inoculated with 10 μL of *B. cinerea* spore suspension (1.0 × 10^5^ spores mL^−1^). Each biological replicate consisted of 20 fruits, and three independent biological replicates were analyzed for each treatment. Data are presented as mean ± SD (*n* = 3). Different letters at the same time point indicate significant differences (*p* < 0.05).

The changes in total phenolics, total flavonoids, and individual phenolic compound contents in cherries across different treatment groups after inoculation are shown in Figure 9. The treatments induced distinct modulation of phenolic metabolism in cherries, with racGR24 promoting pronounced phenolic accumulation and Tis-108 accelerating metabolic turnover. *rac*-GR24 treatment elicited maximal total phenolic content at 24 hpi, achieving a 10.65% greater accumulation than the control. Conversely, Tis-108-treated fruit exhibited depressed phenolic levels, reaching only 94.31% of the control values at the same time point (Figure 9A, *p* < 0.05). After inoculation, the treatments differentially modulated flavonoid accumulation in cherry fruit (Figure 9B). While the control group showed a progressive increase in flavonoid content, the *rac*-GR24 treatment significantly enhanced accumulation, reaching a peak of 11.19 mg rutin equivalents/100 g fresh weight. Conversely, Tis-108 treatment substantially suppressed flavonoid accumulation, maintaining values at just 72.83% of those in the control at terminal inoculation (Figure 9B, *p* < 0.05).

In the present study, four flavonoids (catechin, epicatechin, phloridzin and rutin) and four phenolic acids (protocatechuic acid, neochlorogenic acid, chlorogenic acid and ferulic acid) were identified in sweet cherry fruit (Figure 9). Their accumulation patterns varied significantly among treatment groups. During the initial phase (0–36 h), *rac*-GR24 significantly enhanced catechin, protocatechuic acid, neochlorogenic acid and chlorogenic acid content (Figure 9C,G–I). This stimulatory effect showed progressive attenuation with extended inoculation, ultimately reaching parity with control levels during the late phase (48–60 h).

Epicatechin in control fruit displayed an initial depletion, followed by subsequent accumulation, with concentrations reaching a minimum of 14.19 mg g^−1^ at 24 hpi (Figure 9D). *rac*-GR24 treatment significantly enhanced epicatechin accumulation (*p* < 0.05) while fundamentally altering its temporal profile to a characteristic rise and fall pattern, reaching peak levels at 36 hpi. In contrast, Tis-108 treatment markedly suppressed epicatechin accumulation (*p* < 0.05) yet preserved the decline–increase pattern observed in the control, achieving minimal concentrations at 36 hpi. Excluding 60 h, *rac*-GR24-treated fruit maintained consistently elevated epicatechin concentrations relative to the control throughout the experimental period (*p* < 0.05). Conversely, Tis-108 treatment sustained reduction in epicatechin accumulation compared to control fruit after inoculation (Figure 9D). The control group exhibited a gradual accumulation pattern for rutin, whereas *rac*-GR24 treatment significantly enhanced rutin accumulation, with its levels remaining consistently higher than those of other treatment groups at all time points, except after the early inoculation phase (Figure 9E).

Control fruit exhibited an initial increase, followed by a decline for phlorizin and ferulic acid (Figure 9F,J). In contrast, *rac*-GR24 treatment significantly enhanced the accumulation of both individual phenolic compounds (*p* < 0.01), whereas Tis-108 treatment consistently suppressed their levels below control values throughout inoculation.

### 3.12. Correlation Analysis

**Figure 10:**
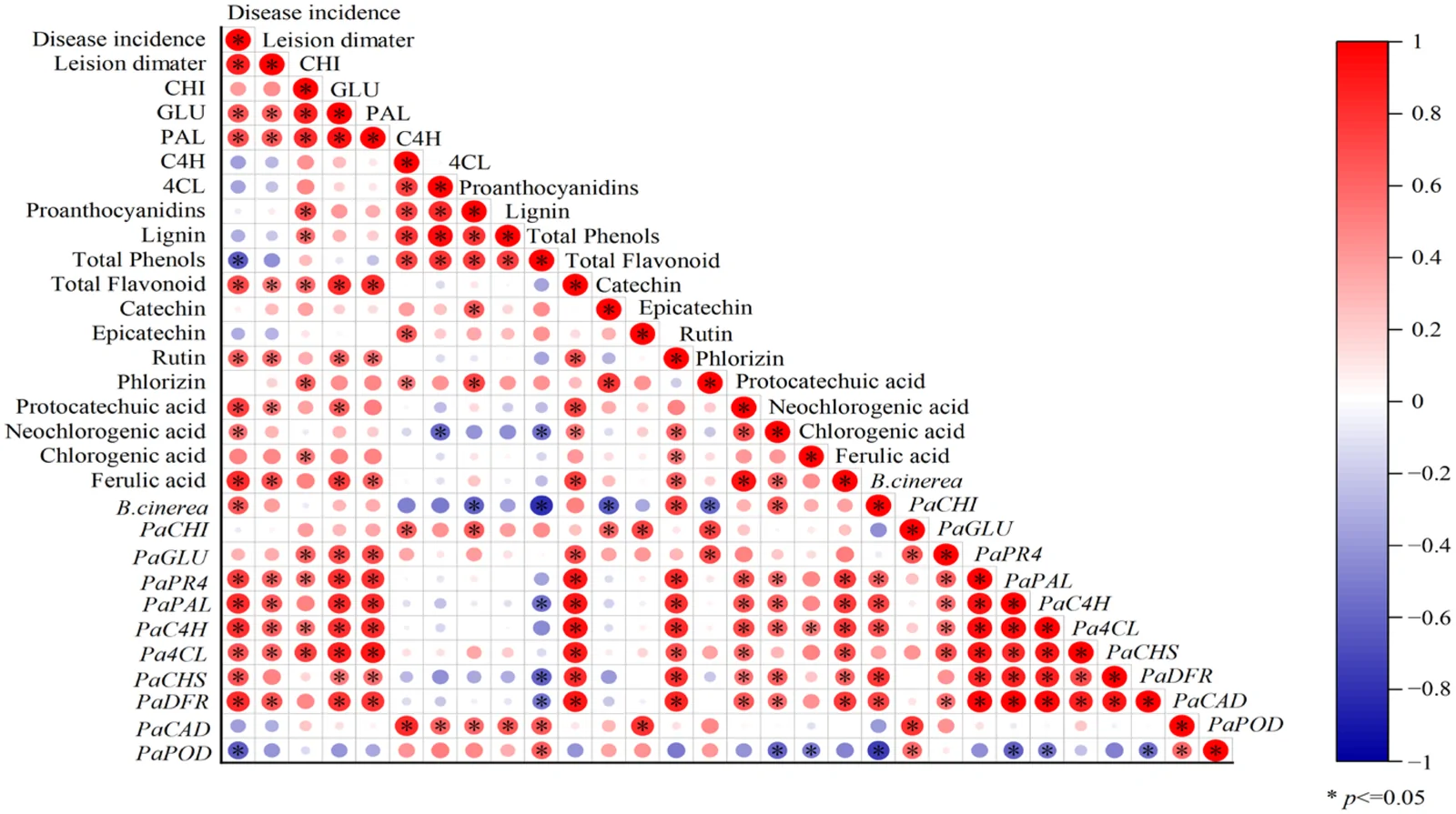
Pearson correlation analysis of disease-related parameters, defense-related enzyme activities, gene expression levels, and metabolite contents in cherry fruits after inoculation. This analysis includes 16 observations across three treatment conditions, measured before treatment (0 h) and at 12, 24, 36, 48, and 60 h after treatment and inoculation. Red (+1) and blue (–1) respectively represent positive and negative correlations among different indicators. An asterisk (*) denotes a statistically significant correlation (*p* < 0.05).

As shown in Figure 10, correlation analysis was performed to evaluate the relationships among disease indicators, defense-related enzyme activities, gene expression levels, and metabolite contents in cherry fruits after inoculation under different treatment conditions. The results revealed that disease-related parameters, including disease incidence, lesion diameter, and relative fungal biomass, were significantly positively correlated with most defense-related enzymes, genes, and metabolites, with total phenolics being the only exception, showing a negative correlation. These correlation patterns suggest that increased activities of defense enzymes, enhanced expression of defense-related genes, and accumulation of defense metabolites may be associated with reduced disease severity in cherry fruits, although further studies are needed to clarify the underlying mechanisms.

## 4. Discussion

Recent studies have highlighted the important role of *rac*-GR24 in regulating plant stress responses such as drought, osmotic, and pathogen stress [44,45,46]. In postharvest crops, *rac*-GR24 has also been reported to regulate cold tolerance [23], quality preservation [21], phenolic metabolism [47], and carbohydrate metabolism [48]. However, its role in resistance against *B. cinerea* in postharvest sweet cherry remains unclear. Therefore, transcriptomic and metabolomic analyses were integrated to investigate the regulatory mechanisms of *rac*-GR24 in phenylpropanoid metabolism and defense responses after sweet cherry inoculation.

In the preliminary concentration–response experiment, low to moderate concentrations of *rac*-GR24 (0.01–0.5 μM) reduced lesion development caused by *B. cinerea*, with 0.1 μM consistently showing the strongest protective effect. In contrast, higher concentrations (≥1.0 μM) unexpectedly promoted lesion development, indicating that the beneficial effect of *rac*-GR24 was restricted to a relatively narrow concentration range (Figure 1). Such concentration-dependent effects are consistent with previous observations that intermediate GR24 concentrations can promote physiological responses, whereas higher concentrations may become inhibitory [49]. The underlying mechanism of this biphasic response remains unclear, but the extensive crosstalk between SLs and other phytohormone pathways provides a possible explanation. SLs interact with jasmonic acid, SA, ET, ABA, and other hormonal pathways, and their effects can depend on the balance among interconnected signaling networks [50]. In particular, *rac*-GR24 has been reported to enhance resistance to *B. cinerea* in *Arabidopsis* by modulating ET- and camalexin-associated defense responses, while SL signaling has also been shown to interact with salicylic acid-mediated disease resistance [46,51]. Therefore, excessive *rac*-GR24 may alter the balance of interconnected hormone signaling or other stress-related physiological processes, potentially reducing the effectiveness of defense responses beyond an optimal signaling threshold. This interpretation is consistent with the concentration-dependent nature of SL responses observed in other plant systems, although the specific mechanism responsible for the enhanced lesion development at high *rac*-GR24 concentrations in postharvest cherry remains to be experimentally determined. Thus, the selection of 0.1 μM *rac*-GR24 in the present study was based on a systematic concentration–response comparison and represents the concentration that produced the most pronounced disease suppression under the experimental conditions used.

### 4.1. Rac-GR24 Enhances Disease Resistance by Activating Defense-Related Enzymes and Genes

Based on the optimized concentration of 0.1 µM identified above, the defense-related responses induced by *rac*-GR24 were further examined. Pathogenesis-related proteins such as CHI and GLU contribute to plant defense by degrading fungal cell walls [52]. Enhanced disease resistance in sweet cherry has been associated with increased CHI and GLU activities and related gene expression under MeJA, luteolin, ABA, and SA treatments [53,54,55,56]. In this study, *rac*-GR24 significantly increased CHI and GLU activities, whereas Tis-108 suppressed these responses (Figure 3A,B). Similarly, *PaCHI*, *PaGLU*, and *PaPR4* expression was markedly induced by *rac*-GR24 but inhibited by Tis-108, particularly during the mid and late stages of infection (Figure 3C–E). These results indicate that *rac*-GR24 signaling positively regulates defense responses in cherry fruit.

Consistent with previous findings in tomato fruit [57], *rac*-GR24 and Tis-108 produced contrasting effects on disease resistance in sweet cherry. Although *rac*-GR24 has been reported to enhance resistance to pathogen infection in tobacco [58], cotton [59], and *Arabidopsis* [46], its role in postharvest disease resistance in sweet cherry remains unclear. Therefore, integrated transcriptomic and metabolomic analyses were conducted to characterize the molecular and metabolic changes associated with *rac*-GR24 treatment and to explore their potential relationship with disease resistance after inoculation.

### 4.2. Multi—Omics Analysis Reveals Metabolic Pathways Affected by Rac-GR24

#### 4.2.1. Rac-GR24 Redirects Phenylalanine Metabolism to Support Defense Precursor Supply

The phenylpropanoid pathway is a central component of plant secondary metabolism, producing phenolic acids, flavonoids, and other defense-related metabolites involved in stress resistance, structural reinforcement, and antioxidant responses [60,61,62]. Changes in phenylpropanoid metabolism have been associated with enhanced disease resistance in postharvest fruit, as reported in MeJA-treated peaches [63], citral-treated citrus [64], and luteolin treated sweet cherries [54].

In the present study, integrated transcriptomic and metabolomic analyses revealed that *rac*-GR24 treatment was associated with substantial changes in phenylpropanoid-related metabolism in sweet cherry fruit. Eighteen DEGs related to phenylalanine, tyrosine, and tryptophan biosynthesis were identified (Figure 5A). The upregulation of *TRP B* was accompanied by changes in tryptophan metabolism, which may have increased the availability of precursors for downstream secondary metabolic pathways, including auxin biosynthesis. Similarly, the altered expression of *OHCmu* and *TAT2* was associated with phenylalanine biosynthesis, whereas increased expression of *PAL* and *copper amine oxidase 1-like* was consistent with enhanced phenylalanine catabolism. Together with the observed changes in phenolic compounds and lignin, these transcriptional and metabolic responses indicate that *rac*-GR24 treatment was associated with a reorganization of phenylpropanoid metabolism during pathogen infection. Similar associations between *rac*-GR24 treatment, phenylpropanoid metabolism, and postharvest disease responses have been reported in apples [65], strawberries [20], sweet oranges [21], and blueberries [66].

#### 4.2.2. Rac-GR24 Activates Phenylpropanoid and Flavonoid Biosynthesis Pathways

Furthermore, *rac*-GR24 significantly upregulated *PAL*, *C4H*, *4CL*, and *CSE* expression, promoting the conversion of p-coumaric acid into downstream hydroxycinnamic acid derivatives and increasing cinnamoyl-CoA accumulation (Figure 5B). Meanwhile, reduced *COMT* expression inhibited sinapic acid and sinapyl alcohol biosynthesis, redirecting metabolic flux toward flavonoid biosynthesis. This was supported by the upregulation of *CHS*, *CHR*, *ANS*, and *DFR*, whereas *PGT1* was downregulated (Figure 5C). In addition, increased *PaCAD* and *PaPOD* expression was associated with enhanced lignin biosynthesis and polymerization, consistent with the higher lignin accumulation observed in *rac*-GR24 treated fruit (Figure 2).

Consistent with previous findings, this study shows that *rac*-GR24 treatment was associated with changes in phenylpropanoid metabolism and enhanced resistance to gray mold in cherry fruit [20,46]. *Rac*-GR24 promoted flavonoid biosynthesis, as indicated by increased *CHR* expression and the accumulation of pinocembrin and naringenin chalcone (Figure 5C). Upregulated *F3H* further facilitated the conversion of naringenin into dihydrokaempferol, which was subsequently metabolized into proanthocyanidin and anthocyanin precursors through *ANS*- and *DFR*-mediated pathways, respectively [67]. Accordingly, metabolites including dihydrokaempferol, luteoforol, homoeriodictyol, naringenin, and pinocembrin accumulated at markedly higher levels under *rac*-GR24 treatment (Figure 5). The coordinated changes in gene expression and metabolite accumulation demonstrate that *rac*-GR24 preferentially directs metabolic flux toward flavonoid biosynthesis, which may contribute to enhanced disease resistance.

### 4.3. SL Pathway Activation in Cherry Fruit Following Pathogen Infection

To verify the regulatory effects of *rac*-GR24 on the SL pathway in cherry fruit, the relative expression levels of *MAX1*, *SMXL6*, and *SMXL7* under different treatments were analyzed by RT-qPCR. The results showed that only *rac*-GR24 treatment markedly induced the expression of *MAX1* and *SMXL7* after inoculation, with a clear time-dependent pattern and peak expression observed at 36 h. *MAX1* functions as a key cytochrome P450 monooxygenase in SL biosynthesis and catalyzes the conversion of SL precursors into bioactive SLs [68,69]. *SMXL6* and *SMXL7* are central negative regulators of SL signaling and participate in SL perception and downstream transcriptional regulation [70]. The coordinated upregulation of these genes under *rac*-GR24 treatment suggests that exogenous SL may promote the activation of both SL biosynthesis and signaling pathways in response to pathogen inoculation. In contrast, Tis-108, a triazole-type inhibitor of SL biosynthesis, suppresses endogenous SL accumulation, thereby weakening the positive regulation of SL signaling on *MAX1* and *SMXL7* expression. These findings indicate that the SL pathway may participate in regulating the response of cherry fruit to pathogen infection and may be closely associated with differences in disease resistance among treatments. Nevertheless, given the inherent limitations of chemical treatments, this interpretation remains tentative and requires further validation using more specific genetic or biochemical approaches.

### 4.4. Coordinated Activation of Phenylpropanoid Metabolism Confers Disease Resistance

To validate the multi-omics results, key components of the phenylpropanoid pathway, including enzyme activities, gene expression, and metabolic intermediates, were further analyzed. As a major secondary metabolic pathway associated with stress resistance, the phenylpropanoid pathway is largely regulated by *PAL*, *C4H*, and *4CL* [61]. *Rac*-GR24 treatment significantly increased the activities of these enzymes and upregulated the expression of *PaPAL*, *PaC4H*, and *Pa4CL* (Figure 7A–F), consistent with the transcriptomic and metabolomic results (Figure 5B). The coordinated changes at the transcriptional and enzymatic levels indicate that *rac*-GR24 treatment is associated with the activation of phenylpropanoid metabolism. Similar effects have also been reported in postharvest blueberries [66] and strawberries [20], suggesting a conserved regulatory mechanism in postharvest fruit. These results indicate that *rac*-GR24 treatment is associated with enhanced disease resistance and changes in phenylpropanoid metabolism and related defense responses.

### 4.5. Accumulation of Phenolics, Flavonoids, and Lignin Mediates Rac-GR24-Induced Resistance

The phenylpropanoid pathway, derived from phenylalanine, produces phenolics and flavonoids that contribute to disease resistance by inhibiting pathogen growth and strengthening cell walls [71,72]. Accumulation of these compounds has been associated with enhanced resistance in postharvest fruit, including MeJA-treated peaches [63], zhinengcong-treated tomatoes [73], and melatonin-treated pears [74]. *Rac*-GR24 has also been reported to promote the accumulation of flavonoids and phenolic acids such as rutin, naringenin, quercetin, kaempferol, chlorogenic acid, and ferulic acid [57].

This study found that *rac*-GR24 treatment was associated with changes in phenylpropanoid metabolism and enhanced disease resistance in cherry fruits. Total phenolics, flavonoids, proanthocyanidins, lignin, phloridzin, and ferulic acid were significantly increased (Figure 8C,F; Figure 9F,J), consistent with their roles in postharvest resistance [75]. *Rac*-GR24 also increased epicatechin accumulation and delayed its peak to 36 hpi, accompanied by enhanced GLU activity (Figure 3B). In addition, rutin levels remained consistently higher after inoculation, while catechin, protocatechuic acid, neochlorogenic acid, and chlorogenic acid accumulated mainly during the early inoculation stage (0–36 h) (Figure 9C,G–I).

The accumulation of phenolics and flavonoids was accompanied by the upregulation of *PaCHS* and *PaDFR* (Figure 8A,B), indicating that *rac*-GR24 activates phenylpropanoid metabolism at both transcriptional and metabolic levels. *Rac*-GR24 also promoted lignin accumulation (Figure 8F) and increased the expression of lignin biosynthesis genes *PaCAD* and *PaPOD* (Figure 8D,E). Meanwhile, transcriptomic and metabolomic analyses showed reduced sinapyl alcohol levels (Figure 5B), likely due to its utilization during lignin polymerization. These results suggest that *rac*-GR24 treatment is associated with changes in phenylpropanoid metabolism and lignin biosynthesis, which coincided with enhanced disease resistance in cherry fruit.

In contrast, Tis-108 treatment was generally associated with reduced levels of these defense-related responses relative to the control, indicating that perturbation of strigolactone-related processes was accompanied by changes in phenylpropanoid metabolism and disease resistance. However, some indicators, including *PAL*, *4CL*, and *PGT1* expression, phenylalanine content, and 4CL activity, showed intermediate levels under Tis-108 treatment (Figure 5). These responses suggest that the effects of Tis-108 treatment on defense-related metabolism were not uniform across all indicators and may reflect differential or compensatory regulatory responses. Similar patterns have also been reported in tomato [57] and strawberry fruit [20] treated with Tis-108.

Correlation analysis further indicated that the transcriptomic data were associated with the analyzed parameters, showing significant positive relationships between the expression of defense-related genes, including *PaCAD*, *PaCHI*, *PaGLU*, and *PaPR4*, and the activities of corresponding enzymes, as well as phenolic and flavonoid accumulation. These results highlight the coordinated changes in phenylpropanoid metabolism and defense responses associated with *rac*-GR24 treatment, which was also associated with enhanced disease resistance in cherry fruit.

## 5. Conclusions

In conclusion, *rac*-GR24 treatment was associated with enhanced disease resistance in postharvest sweet cherries, with 0.1 μM rac-GR24 showing the strongest association with reduced disease incidence and lesion expansion. It upregulated the expression and enzymatic activities of CHI and GLU, two key pathogenesis-related proteins, while differentially modulating *PaPR4* expression. Moreover, it enhanced the expression of genes involved in phenylpropanoid and flavonoid metabolism, including *PAL*, *C4H*, *4CL*, *CHS*, *DFR*, *CAD*, and *POD*, along with the accumulation of defense-related metabolites, including proanthocyanidins, lignin, and individual phenolic compounds. In contrast, Tis-108 exhibited opposite effects across most disease-related parameters and accelerated disease development. Overall, these findings indicate that *rac*-GR24 treatment is associated with changes in defense-related responses and phenylpropanoid metabolism during postharvest gray mold development and support *rac*-GR24 as a sustainable alternative for postharvest disease control.

## Data Availability

The datasets generated and analyzed during the current study are available from the corresponding author upon reasonable request.

## Supplementary Materials

The following supporting information can be downloaded at: https://www.mdpi.com/article/10.3390/foods15213801/s1, Table S1: Primer sequences used for qPCR analysis; Table S2: Sequencing data statistics and QC analysis; Table S3: DEGs FPKM values.
